# Palliative radiotherapy near the end of life

**DOI:** 10.1186/s12904-019-0415-8

**Published:** 2019-03-23

**Authors:** Susan Y. Wu, Lisa Singer, Lauren Boreta, Michael A. Garcia, Shannon E. Fogh, Steve E. Braunstein

**Affiliations:** 1 Department of Radiation Oncology, University of California, San Francisco, 505 Parnassus Ave, L-75, 1600 Divisadero St., H1031, San Francisco, CA 94143 USA; 2 Department of Radiation Oncology, Dana-Farber Cancer Institute and Brigham and Women’s Hospital, Harvard Medical School, 450 Brookline Avenue, Boston, MA 02215 USA

**Keywords:** Palliative radiotherapy, End-of-life care, Cancer

## Abstract

**Background:**

A significant proportion of patients with advanced cancer undergo palliative radiotherapy (RT) within their last 30 days of life. This study characterizes palliative RT at our institution and aims to identify patients who may experience limited benefit from RT due to imminent mortality.

**Methods:**

Five hundred and-eighteen patients treated with external beam RT to a site of metastatic disease between 2012 and 2016 were included. Mann-Whitney U and chi-squared tests were used to identify factors associated with RT within 30 days of death (D_30_RT).

**Results:**

Median age at RT was 63 years (IQR 54–71). Median time from RT to death was 74 days (IQR 33–174). One hundred and twenty-five patients (24%) died within 30 days of RT. D_30_RT was associated with older age at RT (64 vs. 62 years, *p* = 0.04), shorter interval since diagnosis (14 vs. 31 months, *p* <  0.001), liver metastasis (*p* = 0.02), lower KPS (50 vs. 70, *p* <  0.001), lower BMI (22 vs. 24, *p* = 0.001), and inpatient status at consult (56% vs. 26%, *p* < 0.001). Patients who died within 30 days of RT were less likely to have hospice involved in their care (44% vs. 71%, *p* = 0.001). D_30_RT was associated with higher Chow and TEACHH scores at consult (*p* < 0.001 for both).

**Conclusions:**

Twenty-four percent of patients received palliative RT within 30 days of death. Additional tools are necessary to help physicians identify patients who would benefit from short treatment courses or alternative interventions to maximize quality at the end of life.

## Summary

Over half of patients undergoing radiation are treated with palliative intent. This study aims to characterize use of palliative radiation therapy in patients with advanced cancer and identify factors associated with imminent mortality in this patient population.

## Background

More than half of patients treated with radiotherapy (RT) are treated with palliative intent. RT has well-established utility for pain palliation from bone metastases, may be used to improve neurological function or prevent further neurological compromise in patients with brain or spinal cord metastases, and can be used to alleviate symptoms due to obstruction by tumor.

While the response rate to RT in the treatment of bone metastases is high, at approximately 60% [1], the time frame for symptomatic improvement is typically measured in weeks [2–4]. Palliative RT for brain metastases may result in stable or improved neurologic symptoms in about half of patients [5], however is also associated with side effects [6] and may not improve overall survival [7]. Patients undergoing RT at the end of life may not experience symptomatic benefit and may spend a significant proportion of their remaining life expectancy receiving treatment [8]. Time spent on treatment at the end of life may not align with patients’ end of life goals, particularly in the United States where single fraction RT is less commonly utilized. Medicare data suggests that in the United States, almost 8% of patients dying of cancer will receive RT in their last month of life, and almost 20% of these patients will be treated in 10 or more fractions [9, 10].

The purpose of this study is to characterize use of palliative RT in patients with advanced cancer at a single institution and identify factors associated with RT within 30 days of death (D_30_RT).

## Methods

### Patient information

We performed a retrospective single-institution review to identify patients who received external beam RT to a site of metastatic disease between 2012 and 2016. Patients treated with stereotactic radiosurgery (SRS) for limited brain metastases were excluded from this analysis as this represents a highly select group of patients; at our institution, each case is reviewed at a weekly multidisciplinary SRS tumor board and the treatment decision takes into account factors such as patient performance status, control of extracranial disease, and potential systemic therapy options. As our patient list was generated using ICD codes for secondary malignant neoplasms (196–198, C78, and C79), we also excluded patients receiving potentially palliative RT to their primary tumor.

Patient characteristics such as age, gender, primary diagnosis, prior chemotherapy or other systemic treatment, performance status at consult, use of hospice services, and radiation dose/fractionation were abstracted from the medical record. In addition to clinical variables used to calculate the TEACHH and Chow scores described below, we also recorded BMI, as weight loss has been shown to be a poor prognostic sign in patients with cancer [11], and inpatient status at the time of consult [10]. Vital status and date of death were confirmed with our institutional tumor registry. The institutional review board approved this retrospective review. Given the volume of individual patient data and ongoing work, this database has not been made publicly available however is available upon request from the corresponding author.

### Prognostic scores

The TEACCH and Chow models have been described previously [12, 13]. The Chow model of risk factors grouping is simple to use and categorizes patients based on 3 risk factors: non-breast primary, non-bone metastases, and KPS ≤ 60. Group I includes patients with 0–1 risk factors, Group II with 2 risk factors, and group 3 with all three risk factors [14]. The TEACHH model assigns points based on the following risk factors: non-breast or prostate primary, age > 60, ECOG performance status 2–4, liver metastases, hospitalization within 3 months of palliative RT consult, and 2 or more prior palliative chemotherapy courses [12]. Patients with 0–1 risk factors are categorized in group A, 2–4 risk factors in group B, and 5–6 risk factors in group C.

### Statistics

The Shapiro-Wilk test was used to evaluate normality of continuous variables. Mann-Whitney U and Chi-squared tests were used to compare patients who received RT within 30 days of death (D_30_RT) and those who did not. D_30_RT was calculated from the start of RT. Multivariate analysis was used to identify factors associated with D_30_RT. In our exploratory analysis of D_30_RT and inability to complete the prescribed RT course, 16 clinical variables were considered (Table 3); as such we performed a Bonferroni correction and only 2-sided *p*-values less than 0.003 were considered statistically significant. Statistics were performed using IBM SPSS, version 25 (SPSS; Chicago, IL).

## Results

Five hundred and-eighteen patients were included in this analysis. The median age at initial diagnosis was 60 years (interquartile range (IQR) 50–68 years) (Table 1). The median age at final RT course was 63 years (IQR 54–71 years). The median survival time from diagnosis to final RT course was 28 months (IQR 11–53 months). Sixty-six percent of patients (340/518) were Caucasian, while 14% (74/518) were East Asian and 8.7% (45/518) African American. Forty-nine percent of patients (254/518) had metastatic disease at diagnosis. Forty-five percent of patients (231/511) had a KPS > 70 at the time of final RT consult; KPS was not recorded at the time of consultation in 8 patients. Fifty eight percent of patients (289/500) were hospitalized within 3 months of RT consult.

**Table 1:** Patient characteristics

Variable	Median (IQR) or % (n, of 518)	
Age at diagnosis	60 (50–68)	
Percent female	46% (238)	
Race		
White	66% (340)	
East Asian	14% (74)	
African American	8.7% (45)	
Southeast Asian	3.7% (19)	
Asian NOS	3.7% (19)	
Other (includes American Indian, Pacific Islander)	4.1% (21)	
Percent Hispanic	9.1% (47)	
Survival time since diagnosis (months)	28 (11–53)	
Primary diagnosis		
Lung	26% (137)	
Breast	19% (97)	
Prostate	9.7% (50)	
Renal cell	5.8% (30)	
Colorectal	5.8% (30)	
Hepatocellular	3.8% (20)	
Head and Neck	3.5% (18)	
Skin	3.3% (17)	
Other^a^	23% (119)	
Metastatic at diagnosis	49% (254)	
Site of metastases		
Brain	47% (244)	
Lung	55% (284)	
Liver	40% (208)	
Bone only	17% (90)	
BMI last course	24(21–27)	
KPS last consult	60 (50–80)	
KPS > 70	45% (231/511)	
Hospitalization within 3 months of RT consult	58% (289/500)	
TEACHH score^b^		Median survival, months (IQR)
0–1 (Group A)	6.2% (32/450)	6 (2.8–11)
2–4 (Group B)	68% (352/450)	2.2 (1.0–5.0)
5–6 (Group C)	13% (66/450)	1.3 (0.5–2.3)
CHOW group^b^		
I	18% (92/510)	4.7 (2–11)
II	44% (227/510)	2.5 (1.0–5.6)
III	37% (191/510)	1.6 (0.7–2.7)
Hospice involved		
Yes	47% (245)	
No	28% (147)	
Unknown	24% (126)	
Place of death		
Inpatient, acute care	23% (120)	
Home	29% (151)	
Inpatient hospice, non-acute care	10% (52)	
SNF (not hospice)	1.5% (8)	
Unknown	36% (187)	

^a^Includes primary cancer of the liver, bile ducts, esophagus, ovary, pancreas, meninges, endometrium, anus, lymph nodes, CNS, and pleura

^b^Some patients had incomplete information and thus TEACHH or Chow groups could not be calculated (denominators 450 and 510 respectively). Performance status at RT consult was the most commonly missing information, but also hospitalizations within 3 months of RT consult and number of prior palliative chemotherapy courses

The most common primary malignancies were lung (26%, 137/518), breast (19%, 97/518) and prostate (9.7%, 50/518). The most common treatment sites were bone (57%, 293/518) and brain (28%, 146/518) (Table 2). The median number of palliative chemotherapy regimens prior to RT was 1, though the range was quite large (0–13 regimens) (IQR 0–3 regimens).

**Table 2:** Summary of RT

Characteristic	Median (IQR) or % (n, of 518)
Palliative course #	1 (1–2)
Age at RT	63 (54–71)
Prescribed fractions	5 (4–10)
1	17% (89)
2–4	9.6% (50)
5	32% (167)
6–9	3.5% (18)
10	34% (177)
> 10	3.3% (17)
Treatment site	
Bone	57% (293)
Brain	28% (146)
Lung	2.9% (15)
Node	1.7% (9)
Other^a^	11% (55)
Incomplete RT course	12% (63)
Time from start of last RT course to death (days)	74 (33–174)

^a^Includes soft tissue and visceral metastases

The median time from the start of last RT course to death was 74 days (IQR 33–174 days). One hundred and twenty-five patients (24%) died within 30 days of RT. D_30_RT was associated with older median age at initial diagnosis (63 vs. 59 years, *p* = 0.002) shorter interval since diagnosis (14 vs. 31 months, *p* < 0.001), lower median KPS at consultation (50 vs. 70, *p* < 0.001), lower median BMI (22 vs. 24, *p* = 0.001), and inpatient status at consult (56% vs. 26%, *p* < 0.001) (Tables 3 and 4). D_30_RT was associated with higher Chow and TEACHH scores at the time of consult (*p* < 0.001 for both). D_30_RT was associated with a greater likelihood of not completing the prescribed RT course compared to those who lived longer than 30 days following start of RT (42% vs. 6%, *p* < 0.001). Despite poor outcomes, patients who died within 30 days of RT were less likely to have hospice involved in their care (44% vs. 71%, *p* = 0.001). The rate of D_30_RT was not significantly different in patients treated for brain metastases compared to bone metastases (42% vs. 29%, *p* = 0.27), or in patients who were older at the time of RT (*p* = 0.04). On multivariate logistic regression, D_30_RT was associated with older age at diagnosis (*p* < 0.001), older age at RT (*p* < 0.001), and longer interval since initial diagnosis (*p* < 0.001).

**Table 3:** Characteristics of patients and treatment in those who died within 30-days of RT (D_30_RT) and those who did not (D_>30_RT)

	D_30_RT (median (IQR) or % (proportion)^b^)	D_>30_RT (median (IQR) or % (proportion)^b^))	Chi-squared or *p*-value°
Age at diagnosis	63 (52–70)	59 (47–67)	**0.002**
Age at RT	64 (55–73)	62 (52–70)	0.04
Gender, % female	42% (52/125)	47% (184/393)	0.35
% Hispanic	5.8% (7/119)	11% (40/381)	0.15
Survival time (months, diagnosis to RT)	14 (5–38)	31 (14–59)	**< 0.001**
KPS at RT consult	50 (20–70)	70 (50–80)	**< 0.001**
BMI at RT consult	22 (IQR 20–25)	24 (21–27)	**0.001**
Primary diagnosis breast/prostate	18% (22/125)	32% (124/393)	**0.003**
Treatment site			
Bone	53% (66/125)	77% (227/393)	0.35
Brain	34% (43/125)	26% (103/393)	0.09
Lung	4% (5/125)	3% (10/393)	
Other^a^	9%(11/125)	13% (53/393)	
Hospitalization within 3 months of consult	78% (97/125)	51% (192/375)	**< 0.001**
Metastatic at diagnosis	50% (62/125)	50% (191/388)	0.94
Sites of metastases			
Non-bone	90% (112/125)	80% (315/392)	0.08
Brain	51% (63/124)	47% (181/387)	
Lung	62% (78/125)	53% (206/390)	
Liver	50% (62/125)	38% (146/386)	
Prescribed fractions^c^	5 (3–10)	5 (4–10)	0.14
TEACHH Group			**< 0.001**
A	1.6% (2/124)	9% (30/326)	
B	74% (92/124)	80%(260/326)	
C	24%(30/124)	11%(36/326)	
Chow group			**< 0.001**
I	3% (5/124)	23% (87/386)	
II	41% (51/124)	46% (176/386)	
III	55% (68/124)	32% (123/386)	
Inpatient consult	56% (70/125)	26% (103/393)	**< 0.001**
Hospice involved	44% (54/122)	71% (191/270)	**< 0.001**

°values in bold are statistically significant given our adjusted α of 0.003

^a^Includes soft tissue and visceral metastases

^b^Denominators reflect missing data and thus are not all 125 (D_30_RT) or 393 (D_>30_RT)

^c^Prescribed fractions was not considered in the Bonferroni correction, as this is a decision made by the treating radiation oncologist based on clinical variables

**Table 4:** Risk of death within 30 days based on clinical variables

Clinical variable	Risk of death within 30 days, % (proportion)
Age (years) at RT	
> 60	26% (76/294)
> 70	30% (42/142)
> 80	30% (9/30)
KPS < 70 at RT	33% (91/280)
Treatment site	
Bone	23% (67/294)
Brain	29% (42/145)
Hospitalized within 3 months of RT consult	34% (97/289)
TEACHH group	
A	6% (2/32)
B	26% (92/352)
C	45% (30/66)
CHOW group	
I	5%(5/92)
II	22% (51/227)
III	36% (68/191)

Overall, 12% of patients (63/518) did not complete their final RT course. Patients who did not complete radiation were more likely to be inpatients at the time of RT consultation (19% vs. 9%, *p* = 0.001) or have been hospitalized within 3 months of RT (16% vs. 8%, *p* = 0.005). Patients who did not complete treatment were more likely to have a KPS < 70 than those who completed treatment (84% vs. 51%, *p* < 0.001). Patients with a BMI < 25th percentile were less likely to complete RT than those with a BMI ≥ 25th percentile (62% vs. 76%, *p* = 0.02). Patients who did not complete RT were prescribed more fractions than those who completed RT (median 8 vs. 5 fractions, *p* = 0.001). Patients who did not complete RT had a shorter period from last RT to death compared to those who did complete treatment (median 18 vs. 73 days, *p* < 0.001). Patients unable to complete their last RT course were more likely to be in TEACHH group C (24% vs. 11%, *p* < 0.001) and Chow group III (55% vs. 32%, *p* < 0.001). Inability to complete RT was not different in those receiving RT to brain vs. bone metastases (*p* = 0.08). On multivariate logistic regression, inability to complete RT was associated with lower KPS (*p* < 0.001) and metastatic disease at diagnosis (*p* = 0.001).

Increased hospice enrollment was associated with a longer interval since diagnosis (28 months vs. 21 months, *p* = 0.04). Hospice was less likely to be involved when inpatients were evaluated for RT compared to outpatients (31% vs. 42%, *p* = 0.02). There was no association between age at diagnosis, age at RT, TEACHH or Chow score, or KPS and hospice involvement. Patients enrolled in hospice were less likely to die in a hospital setting (6.2%) but rather at home (67%) or in a non-acute care inpatient setting (27%, inpatient hospice unit or skilled nursing facility) compared to those not enrolled in hospice (81% in a hospital, 13% at home, 6% non-acute care inpatient) (*p* < 0.001).

## Discussion

Radiotherapy can be very effective at palliating symptoms, however does not take effect immediately and can entail multiple clinic visits over the treatment course. As such, palliative RT should be used thoughtfully in patients with advanced cancer, with special attention to intent, fractionation pattern, and goals of care for each patient. This study applies validated, cancer-specific prognostic tools to patients undergoing palliative radiotherapy to sites of metastatic disease at a large academic institution, and characterizes patients who received radiation within 30 days of death. This study also highlights clinical factors associated with incomplete RT courses, which may be viewed as a quality indictor for selecting an appropriate dose and fractionation regimen in appropriate patients.

Almost one-quarter of patients receiving palliative RT in our series were treated within their last 30 days of life, a rate higher than many published series [8, 9, 15–17]. This is likely in part due to the fact that our analysis was restricted to patients receiving palliative RT to metastases (i.e. no palliation of the primary tumor) and excluded patients treated with Gamma Knife radiosurgery, thereby selecting for patients with greater intracranial metastatic burden and/or poorer performance status. It has been shown that D_30_RT may be higher in patients with more advanced disease at diagnosis [18] or with certain primary tumors, particularly lung. Kapadia et al. demonstrated that in patients with non-small cell lung cancer, those who were metastatic at diagnosis were twice as likely to undergo radiation at the end of life [18]. Even among patients with metastatic disease, those with multiple metastases were 75% more likely to undergo radiation within 2 weeks of death than patients with a single site of metastatic disease. Murphy et al. demonstrated that patients with primary lung cancer had an odds ratio for death within 1 month of completing RT of 3.8 compared to patients with prostate cancer [19]. Consistent with our data, the rate of D_30_RT may be high in patients receiving palliative RT to bone or brain metastases (26 and 23%, respectively) [10] [20]. However, a very low rate of D_30_RT is not necessarily ideal, as this may reflect treatment being withheld from patients who may otherwise benefit from palliative-directed RT. On the other end of the spectrum, a high D_30_RT may suggest overly aggressive treatment or selection of RT fractionation regimens that are too protracted in duration and misaligned with patient-specific needs.

Forty-two percent of patients who received RT within 30 days of death in our cohort did not complete their planned RT course, consistent with the literature [21]. This may be due, in part, to the fact that prognostication at the end of life is a difficult task and physicians are often overly optimistic [22, 23]. Several tools have been developed to assist in estimating life expectancy. The palliative prognostic index uses palliative performance status, which is strongly correlated with, and can be used interchangeably with, KPS, oral intake, and clinical symptoms such as dyspnea, delirium, and edema to estimate life expectancy in patients receiving palliative care [24, 25], and performs comparably to similar scores that also take into account white blood cell count, lymphocyte percentage, or delirium in cancer patients [26]. A nomogram has also been created that includes time since diagnosis, performance status, albumin, lactate dehydrogenase, and lymphocyte count to predict 15, 30, and 60-day survival [27].

These tools, however, do not evaluate prognosis using *cancer* specific characteristics. The TEACHH score and Chow model are two prognostic tools that have been developed to predict life expectancy in patients with advanced cancer [12, 13]. Both take into account KPS and primary diagnosis; the Chow model also incorporates non-bone metastases while the TEACHH score includes prior chemotherapy, recent hospitalizations, and specifically hepatic metastases. The TEACHH score categorizes patients into three groups (A, B, and C) with distinct survival times from the start of RT (19.9 months, 5 months, and 1.7 months, respectively) [12]. The Chow “number of risk factors” model categorizes patients into three groups (I, II, and III) with median survival times of approximately 15, 6.5, and 2.5 months respectively [13]. A recently published "NEAT" model is similar to the TEACHH score but also incorporates albumin levels to yield four prognostic groups with median survivals of 24.9, 14.8, 4.0, and 1.2 months [14].

In our cohort, median survival was shorter than estimated across all TEACHH and Chow groups (Table 1). This may reflect use of palliative RT earlier in the disease course among the TEACHH cohort, with a shorter time from diagnosis to RT consult (1.8 months, calculated as the sum of time from diagnosis to metastasis and from metastasis to RT consult), compared to 28 months in our cohort. Patients in our cohort were also more likely to have received prior palliative RT than patients in the TEACHH cohort (44% vs. 12.5%). Compared to the Chow training set, our cohort had a substantially lower percentage of patients with bone-only metastases (17% vs. 29%), which may translate into a more significant disease burden and thus poorer prognosis in our patients. As only 45% of patients (30/66) in TEACHH group C and 36% of patients (68/191) in Chow group III died within 30 days of RT, the integrated prognostic tools currently available do not appear sufficiently specific to identify patients at risk for imminent death at the time of RT consultation.

In the United States, there is a tendency to prescribe more protracted treatment regimens in patients with longer anticipated survival [28]. Initial concern regarding durability of control following short course RT may have stemmed from higher re-treatment rates seen following single-fraction RT in RTOG 9714 [29], however the Dutch Bone Metastases Study showed that re-irradiation occurred at a higher rate among non-responders and at lower pain scores in the cohort that received single fraction RT compared to the cohort that received multi-fraction RT, despite similar overall response rates, time to, and duration of response [3]. This suggests that higher retreatment rates after single fraction RT may be due to physician views on the safety of retreatment.

A large body of evidence has demonstrated that single fraction RT courses are as effective as more protracted courses with regard to onset of symptomatic improvement, duration of relief, proportion of patients experiencing improvement, and subsequent quality of life in patients with bone metastases [1, 30, 31]. Similarly, no overall survival benefit has been demonstrated with longer RT courses in the treatment of malignant cord compression or brain metastases [7, 32, 33]. However, a survey of practicing members of the American Society of Radiation Oncology suggests the most common palliative fractionation pattern in the United States remains 30 Gy in 10 fractions; single-fraction treatment is more common among those practicing in Canada, Australia, and New Zealand [34]. In a survey of radiation oncologists practicing within the Veterans Healthcare Administration, physicians who had been in practice for more than 10 years were less likely to offer single fraction RT compared to those with fewer years in practice (63% vs. 90%, *p* = 0.01) suggesting there may be shifts in practice patterns over time [35]. Of note, this survey also found that those who had ever worked in private practice were less likely to offer single fraction RT (64% vs. 88%, *p* = 0.03), suggesting that practice patterns may be influenced by practice setting.

Patients receiving RT at the end of life are increasingly receiving more advanced treatment modalities, with a decrease in the proportion receiving 2D RT from 75 to 33% from 2000 to 2009 [36]. Use of 3D RT increased from 27 to 59%, and use of IMRT increased from 0 to 6.2% over the same period. As patients live longer with advanced cancer, and potentially receive more palliative RT courses, there may be indications for such techniques, including retreatment or treatment in close proximity to prior fields. However more advanced planning techniques require more planning and quality assurance time, which is already limited for patients with poor prognosis.

When used appropriately, palliative RT in patients with advanced cancer may relieve symptoms and preserve quality of life. However radiotherapy remains a local treatment. Patients with advanced cancer suffer from a broad range of symptoms that RT may not be able to address, such as depression, anxiety, or anorexia/cachexia. Furthermore RT, depending on the treatment site, may cause symptoms that can, in turn, diminish quality of life, including fatigue, nausea, or xerostomia. As such, it is critical that patients receive palliative care services early in their disease course, in parallel with disease directed care. Palliative care should start with the primary interdisciplinary oncology team, with referrals to palliative care specialists if patient needs are complex; this approach is supported by the NCCN [37], WHO [38], and ASCO [39]. There is level I evidence supporting early integration of palliative care with regard to patient reported quality of life [40], as well as duration of life [41].

Earlier integration of palliative care fits with a growing notion of primary palliative care—that is, a fundamental level of palliative care proficiency that should be expected of all clinicians, which can be augmented by palliative care specialists as needed [42]. Indeed, given almost half of RT courses are palliative in nature, radiation oncologists should also consider themselves palliative care providers and co-manage symptoms with other providers. Furthermore, as palliative care needs vary substantially throughout a patient’ disease course [43], they should be reassessed at regular intervals in all cancer patients [39].

This study is limited in that data was obtained retrospectively and may be incomplete, particularly for patients who were seen prior to the transition to electronic medical records or who received care at other institutions. In particular, data regarding prior chemotherapy was often incomplete; while we typically had records documenting the regimen, we often lacked the total number of cycles received. Documentation of the specific indication for palliative RT was inconsistent and highly heterogeneous, which made further analysis difficult. Furthermore, due to the retrospective nature of the data, our information regarding symptomatic improvement and quality of life in patients undergoing palliative radiation at the end of life is limited. Patients treated at our institution may have more advanced disease than those treated at other institutions, particularly patients enrolled in Phase I trials or seen in the inpatient setting. The exploratory nature of this analysis must also be emphasized. We have attempted to reduce the risk of Type I error using a Bonferroni correction, however it remains that these analysis were conducted without a specific predetermined hypothesis. Additionally, a significant proportion of patients not enrolled in hospice were being followed by palliative care services. We were unable to more thoroughly assess patterns of palliative care referrals or quantify use of palliative care services in this cohort due to changes in referral codes over time. However it is likely that end-of-life and goals-of-care discussions were occurring more often than it would seem solely based on the rate of hospice enrollment.

## Conclusion

A substantial proportion of patients with advanced cancer undergo palliative RT within 30 days of death, suggesting that there remains a great deal of work to be done to improve the quality of care delivered at the end of life. Palliative RT must align with patient-directed goals of care, and offer maximal palliation while maintaining quality of remaining life. Prognostication for individual patients with advanced cancer continues to be is quite difficult, and the current tools available are not specific for patients at imminent risk of death. All patients with advanced cancer should receive multidisciplinary palliative care from their treating oncologists and, as needs become more complex, palliative care specialists are of great value.

## Abbreviations

ASCO: American Society for Clinical Oncology
BMI: Body mass index
D_30_RT: Radiotherapy within 30 days of death
Gy: Gray
ICD: International Classification of Disease
IQR: Interquartile range
KPS: Karnofsky performance status
NCCN: National Comprehensive Cancer Network
RT: Radiotherapy
RTOG: Radiation Therapy Oncology Group
SRS: Stereotactic radiosurgery
WHO: World Health Organization
